# Perfusion Index Derived from a Pulse Oximeter Can Detect Changes in Peripheral Microcirculation during Uretero-Renal-Scopy Stone Manipulation (URS-SM)

**DOI:** 10.1371/journal.pone.0115743

**Published:** 2014-12-26

**Authors:** Ho-Shiang Huang, Chun-Lin Chu, Chia-Ti Tsai, Cho-Kai Wu, Ling-Ping Lai, Huei-Ming Yeh

**Affiliations:** 1 Department of Urology, National Cheng Kung University Hospital, College of Medicine, National Cheng Kung University, Tainan, Taiwan; 2 Institute of Biomedical Engineering National Taiwan University, Taipei, Taiwan; 3 Department of Anesthesiology, National Taiwan University Hospital Yun-Lin Branch, Yun-Lin, Taiwan; 4 Division of Cardiology, Department of Cardiology, National Taiwan University Hospital, Taipei and Yun-Lin, Taiwan; 5 Department of Anesthesiology, National Taiwan University Hospital, Taipei, Taiwan; UNIFESP Federal University of São Paulo, Brazil

## Abstract

**Background:**

The objective of this study was to test the effect of removal of a ureteral obstruction (renal calculus) from anesthetized patients on the perfusion index (PI), as measured by a pulse oximeter, and on the estimated glomerular filtration rate (eGFR).

**Patients and Methods:**

This prospective study enrolled 113 patients with unilateral ureteral obstructions (kidney stones) who were scheduled for ureteroscopy (URS) laser lithotripsy. One urologist graded patient hydronephrosis before surgery. A pulse oximeter was affixed to each patient's index finger ipsilateral to the intravenous catheter, and a non-invasive blood pressure cuff was placed on the contralateral side. Ipsilateral double J stents and Foley catheters were inserted and left indwelling for 24 h. PI and mean arterial pressure (MAP) were determined at baseline, 5 min after anesthesia, and 10 min after surgery; eGFR was determined at admission, 1 day after surgery, and 14 days after surgery.

**Results:**

Patients with different grades of hydronephrosis had similar age, eGFR, PI, mean arterial pressure (MAP), and heart rate (HR). PI increased significantly in each hydronephrosis group after ureteral stone disintegration. None of the groups had significant post-URS changes in eGFR, although eGFR increased in the grade I hydronephrosis group after 14 days. The percent change of PI correlates significantly with the percent change of MAP, but not with that of eGFR.

**Conclusion:**

Our results demonstrate that release of a ureteral obstruction leads to a concurrent increase of PI during anesthesia. Measurement of PI may be a valuable tool to monitor the successful release of ureteral obstructions and changes of microcirculation during surgery. There were also increases in eGFR after 14 days, but not immediately after surgery.

## Introduction

Modern anesthetic practice places increasing emphasis on changes in microcirculation following the initiation of newly developed devices, and this has led to improved organ perfusion and reduced post-operative morbidity [1]. The oximeter probe (Masimo Corp., Irvine, CA, USA) projects infrared light through the tissue bed of the finger tip and can assess peripheral perfusion. Recent studies have used this instrument to monitor peripheral vascular tone in pregnant woman and predict hypotension after spinal anesthesia [2], and to provide early prediction of successful brachial plexus block [3]. Based on previous studies, a change in the perfusion index (PI) is a rapid indicator of change in peripheral microcirculation, and this information may help anesthetists to make more appropriate treatment decisions [4]–[6].

Hydronephrosis can alter tubuloglomerular feedback and may lead to hypertension [7]. A 1974 animal study reported a decline in renal hemodynamics when experimental animals underwent 24 h of unilateral ureteral obstruction [8]. Moreover, Harris *et al.* [9] showed loss of excretory function in the post-obstructive kidney when tested by saline loading after release of 24-hocclusion. These results suggest that the impaired diuretic/natriuretic responses in the post-obstructive kidney might be due to reduced hemodynamics, and possibly to increased renal sympathetic nerve activity [10]. These effects change the pressure inside the glomerular capillaries [11], and may lead to a change in microcirculation.

In the present study, we used the oximeter probe to test the hypothesis that release of a ureteral obstruction (renal calculus) improves renal hemodynamics and microcirculation, and that these are promptly indicated by increases in the PI. We also examined the effect of removal of ureteral obstructions on the post-operative estimated glomerular filtration rate (eGFR).

## Methods

This prospective study was approved by the National Taiwan University Hospital Ethics Committee (201205117RIC). (S1 and S2 Txt) One hundred and twenty-six patients diagnosed with ureteral stones were admitted to our department (National Taiwan University Hospital, Taipei, Taiwan) for ureteroscopy (URS) and laser lithotripsy between September 2012 and June 2013. One hundred and thirteen patients were ultimately enrolled. (S3 and S4 Txt) The exclusion criteria were: morbid obesity (BMI>40 kg/m^2^), peripheral arterial disease, usage of vasoactive agents, and contraindications to intravenous anesthesia. Although endothelial dysfunction may interfere the amplitude and velocity of changes in PI, we observed changes in PI for more than 5 min after stone disintegration, so this delayed effect can be ignored. There were 33 women and 80 men, the mean age was 53.7 years (range: 20–79 years), and American Society of Anesthesiologists Scores (ASA Scores) were between I and III. The grades of pre-operative hydronephrosis were classified according to Goertz JK and Lotterman S: grade I was defined as enlargement of the calyces with preservation of the renal papillae, grade II as rounding of the calyces with obliteration of the renal papillae, and grade III as calyceal ballooning with cortical thinning [12].

### Study protocol

We obtained written informed consent from patients for enrollment at the pre-anesthesia visit on the day before surgery. No medications were given to patients before entering the operating room (OR). A urologist graded the severity of hydronephrosis by renal echography at the out-patient department (OPD) visit. After arrival at the OR, each patient was placed in a supine position and fitted with a non-invasive blood pressure cuff, a 3-lead electrocardiogram, and a pulse oximeter probe (Masimo Corp., Irvine, CA, USA) that was attached to the index finger on the ipsilateral side of intravenous catheter. After the signal stabilized, an average of five consecutive readings was recorded (baseline). After reaching a steady depth of anesthesia, the PI was recorded at 5-minintervals (pre-URS). A 6F/7.5F semi-rigid URS (Richard Wolf Medical Instruments Corporation, Vernon Hills, IL) was then introduced into the ureter retrogradely along with a safety guide wire to locate the ureteral stone. Then, the stone was disintegrated by use of a Holmium:YAG laser (Odyssey, Convergent Laser Technologies, Alameda, CA,USA). After stone disintegration, readings were defined as post-URS. Ureteral patency was re-examined by URS assure that there was no ureteral injury caused by laser lithotripsy. All procedures involved insertion of a 28F ureteral double J stent into the operated side. The success rate was 100% and the 16F Foley catheter remained in place for one day.

PI was calculated by measurement of the constant amount of light absorbed by non-pulsatile blood and other tissue (DC) and the variable amount of light absorbed by pulsating arterial inflow(AC)and use of the following equation: PI = (AC/DC)×100%. Anesthesia was administered as target-controlled infusion of propofol, and the blood concentration target was 5 µg/mL. Fentanyl was given at a concentration of 1 µg/kg body weight. Entropy values were maintained between 40 and 60 with adjustment of the target concentration of propofol by 0.5 µg/mL to achieve a steady state of sedation. The ambient temperature was 22°C and intravenous fluids (37°C) were infused at a rate of 500 mL/h.

### Measurement of Serum Creatinine

Serum creatinine (SCr) was measured three times in all patients: at baseline (upon admission), one day after URS laser lithotripsy, and 14 days after URS laser lithotripsy. The eGFR was calculated as: 186×(Serum Cr)−1.154×(age)−0.203×(0.742 if female)×(1.210 if African- American) [13], [14].

### Data analysis

Statistical analysis employed SPSS ver. 19, and data are presented as means ±SDs. The effect of sex and ASA score for patients with different hydronephrosis grades were examined by chi-square tests. Numerical data that had normal distributions and patient data were analyzed by one-way analysis of variance (ANOVA). Changes of variables in each hydronephrosis group were analyzed by repeated measure ANOVA. If there was a significant difference, a post-hoc Turkey HSD test was used to test for differences between groups. Pearson's correlation coefficient (γ) was used to assess the correlation between clinical variables or baseline parameters and the percent change in mean arterial pressure (MAP), PI, and eGFR. The interquartile range (IQR) was plotted relative to PI and MAP over different time periods. A *p*-value less than 0.05 was considered statistically significant.

## Results

Table 1 shows the demographic data of patients with grade I, II, and III hydronephrosis. The 3 groups were similar with regard to age, sex distribution, and ASA score. We recorded MAP, HR, and PI during three time periods (baseline, pre-URS, and post-URS), and there were no significant differences among the groups at each time. The percentage change of PI from baseline to the pre-URS period (effect of anesthesia) and the pre-URS period to the post-URS period (effect of URS) also indicated no significant differences among the groups. eGFR among these groups also showed no difference between pre-URS and post-URS estimates. However, the eGFR at 14 days after surgery was significantly greater in patients with grade I hydronephrosis (*p* = 0.008).

**Table 1:** Demographic data and the degree of hydronephrosis.

Hydronephrosis variables	Grade I (n = 53)	Grade II (n = 41)	Grade III (n = 19)	*P* value
Age (Yr)	52 (25–73)	54 (20–79)	56 (33–75)	0.46
Gender				
Male/Female	35/18	33/8	12/7	0.23
eGFR(mL/min/1.73 m^2^)				
Pre-URS	86.6+/−24.9	77.4+/−27.2	82.4+/−27.0	0.24
Post-URS	85.8+/−24.6	77.5+/−23.7	83.1+/−27.9	0.27
14 days	91.7+/−25.9	79.4+/−27.6	83.0+/−30.1	*0.008*
ASA score I/II/III	10/39/4	6/27/8	2/14/3	0.45
PI				
Baseline	1.8+/−1.3	1.6+/−1.1	2.0+/−1.2	0.56
%change	309+/−54	250+/−29	195+/−34	0.326
Pre-URS	4.9+/−2.4	4.5+/−2.1	4.9+/−2.2	0.84
%change	66.8+/−14	67.3+/−25	50.2+/−14	0.86
Post-URS	6.8+/−2.4	6.5+/−2.7	6.6+/−2.6	0.83
MAP (mm Hg)				
Baseline	104.6+/−11.8	108.9+/−11.4	105.9+/−10.1	0.19
Pre-URS	91.4+/−11.6	94.3+/−10.9	93.2+/−13.4	0.46
Post-URS	90.1+/−11.5	93.1+/−14.0	95.3+/−19.2	0.31
HR (beats min^−1^)				
Baseline	72.6+/−12.9	71.3+/−14.5	77.1+/−12.4	0.30
Pre-URS	73.6+/−11.8	75.1+/−12.2	76.6+/−14.1	0.62
Post-URS	76.9+/−12.3	79.1+/−13.9	79.4+/−14.0	0.65

Legend 1.No significant difference among these groups. Only eGFR 14 days after URS showed significantly different.

Footnote 1.abbreviation: eGFR, estimated glomerular filtration rate; ASA, American Society of Anesthesiologists; MAP, Mean arterial pressure; HR, heart rate; URS, ureteroscopy.

Table 2 shows the changes in PI, MAP, and eGFR in the 3 hydronephrosis groups. Repeated measures ANOVA indicated that the PI values of each hydronephrosis group increased significantly over time (*p*<0.05 for all). In addition, Turkey HSD tests of the PI values indicated significant differences between baseline and pre-URS values, and between pre-URS and post-URS values (*p*<0.01 for all).The MAP also declined significantly in all 3 hydronephrosis groups (*p*<0.05 for all), but the difference between pre-URS and post-URS values were not significant when compared with post hoc test. The increase of eGFR was significant only in patients with grade I hydronephrosis (*p*<0.05), and the Turkey HSD test indicated significant differences only between pre-URS values and values at 14 days after surgery (*p*<0.05).

**Table 2:** The changes in PI, MAP, and eGFR in the 3 hydronephrosis groups. (compared with repeat measurement ANOVA and then Turkey HSD test each other).

		Grade 1	Grade 2	Grade 3
Parameter		n = 53	n = 41	n = 19
PI	Baseline	1.8	1.6	2.0
	Pre-URS	4.9	4.5	4.9
	Post-URS	6.9	6.5	6.6
	p value	p<0.05^1^	p<0.05^1^	p<0.05^1^
MAP	Baseline	104.6	108.9	105.9
	Pre-URS	91.4	94.3	93.2
	Post-URS	90.1	93.1	95.3
	p value	p<0.05^2^	p<0.05^2^	p<0.05^2^
eGFR	Pre-URS	86.6	77.4	82.4
	Post-URS	85.8	77.5	83.1
	14 days	91.7	79.4	83.0
	p value	p<0.05^3^	p = 0.57	p = 0.96

Legend 2. PI in three hydronephrosis groups increase significantly after anesthesia and increases further after releasing urinary obstruction.

Footnote 2. ^1^Baseline vs Pre-URS vs Post-URS PI in each hydronephrosis group: all significantly different when compared each other by Turkey HSD test (p<0.01);

2 Baseline vs Pre-URS, Baseline vs Post-URS MAP in each hydronephrosis group: significantly different when tested by Turkey HSD test (p<0.01), Pre-URS vs Post-URS MAP: not significantly different; ^3^Pre-URS vs 14 days, Post-URS vs 14 days eGFR in grade I hydronephrosis: significantly different (p<0.05).

Table 3 shows the relationships between percent changes in MAP, PI, and eGFR after stone disintegration for all patients which showed the effect of releasing obstruction on each parameters. The change ratios of MAP and PI were not significantly correlated with baseline PI, MAP, or eGFR. However, the percent change of eGFR14 days after surgery was negatively correlated with baseline eGFR (γ = −0.32, *p*<0.05). The change ratios of PI, MAP, and eGFR were also not significantly different in patients with different grades of hydronephrosis (one-way ANOVA). However, the change ratio of PI was significantly and inversely correlated with the change ratio of MAP (γ = −0.30, *p*<0.05).

**Table 3:** Relationships between baseline clinical parameters and the percent changes in MAP, PI, eGFR after stone disintegration.

	% MAP change^#^		% PI changes^+^		% eGFR changes^↑^	
	Γ	P value	γ	P value	γ	P value
Baseline MAP (mmHg)	−0.12	0.17	0.13	0.14	−0.12	0.20
Baseline PI	−0.01	0.87	−0.13	0.17	0.00	0.98
Baseline eGFR (mL/min/1.73 m^2^)	0.02	0.77	0.17	0.06	−0.32	***<0.05***
Grade of hydronephrosis		0.52		0.86		0.56
			γ		P value	
% MAP change^#^ % PI changes^+^			−0.30		***<0.05***	
% PI changes^+^ % eGFR changes^↑^			−0.10		0.33	
% eGFR changes^↑^ % MAP change^#^			0.09		0.30	

Legend 3. Percent changes in MAP, PI, and eGFR have no correlation with baseline MAP, PI, and eGFR. There was also no significant difference in percent change in PI, MAP, and eGFR between each grade of hydronephrosis. Percent change of MAP has negative correlation with percent change of PI.

Footnote 3. ^#^Percent change of MAP: (post-URS MAP-pre-URS MAP)/pre-URS MAP; ^+^Percent change of PI: (post-URS PI-pre-URS PI)/pre-URS PI; ^↑^Percent change of eGFR: (14 days eGFR-pre-URS eGFR)/pre-URS eGFR.

**Figure 1:**
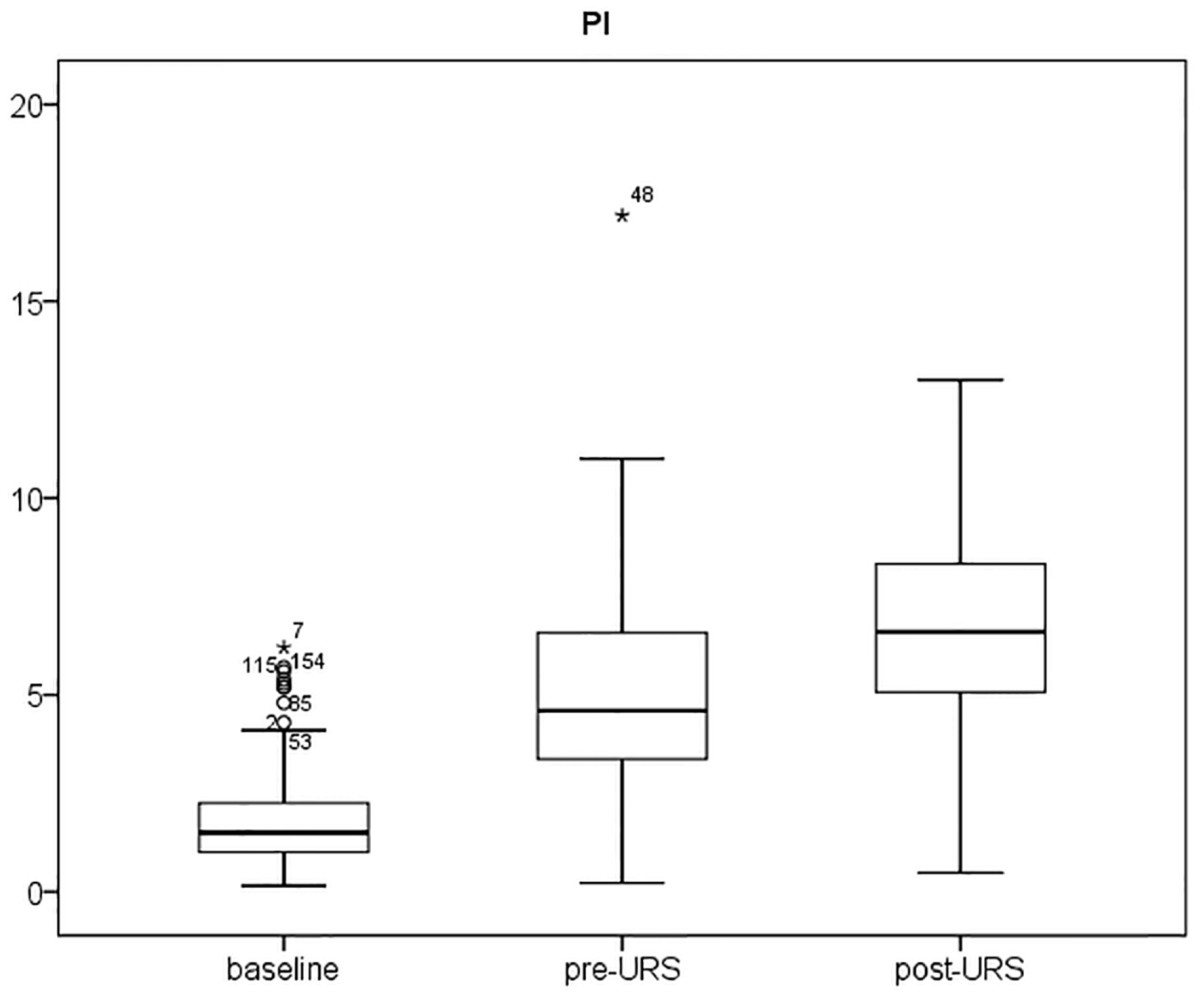
Interquartile range (IQR) of Perfusion Index (PI) in patients with Grade I, II, and III hydronephrosis. The PI increased after induction of anesthesia, and increased further after stone disintegration.

**Figure 2:**
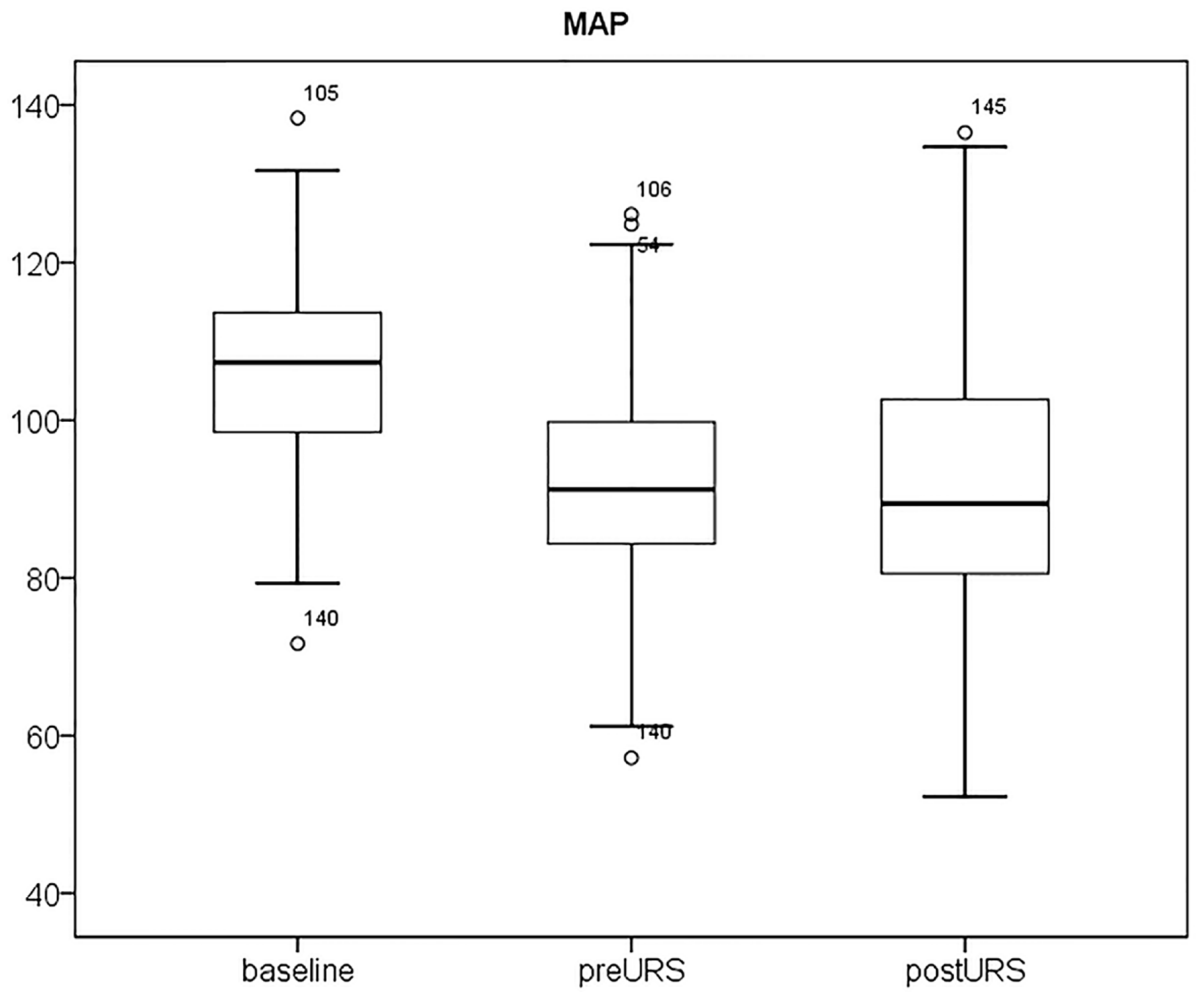
Interquartile range (IQR) of mean arterial pressure (MAP) in patients with Grade I, II, and III hydronephrosis. The MAP decreased after induction of anesthesia, but there were no further changes during stone evacuation.

Figs. 1 and 2 show the PI and MAP at the 3 time periods. PI increased after intravenous anesthesia and increased further after stone disintegration. MAP decreased after induction of anesthesia, but there was no further change during stone evacuation.

## Discussion

Calculation of the PI by pulse oximetry provides a measure of changes in peripheral perfusion in the finger. In particular, the PI can predict postoperative shivering [15] and is an early predictor of survival after resuscitation [16]. Based on these previous studies, we conclude that changes in the PI are related to changes in peripheral microcirculation, and these are correlated with vascular status, sympathetic reactions, and function of the circulatory system.

In the present study, anesthesia and surgery of patients with renal calculi increased the measured PI in all hydronephrosis groups. Propofol relaxes vessel tone and decreases sympathetic reactions, resulting in an increase of the PI [17], and this is the likely cause of the 195–309% increase of PI in our patients after anesthesia. However, patients in all 3 of our hydronephrosis groups experienced a further 50.2–67.3% increase of PI after ureteral stone destruction, even though the level of sedation remained steady. Moreover, while PI increased after stone destruction, the MAP did not change significantly. This phenomenon was first discovered in this study, and it supports our hypothesis that release of ureteral obstruction by URS and laser lithotripsy increases microcirculation during anesthesia and that these changes can be measured by calculation of PI from a pulse oximeter. Our patients had no active changes in sympathetic tone when under anesthesia, and changes in body temperature can be ignored because of the short duration of surgery. The sudden drop of intra-renal pressure following URS laser lithotripsy is a key factor explaining the subsequent increase of the PI. Stone disintegration and the reestablishment of ureteral patency by an indwelling double-J catheter affects the circulatory system, possibly by free radicals and release of cytokines [18], [19], and this leads to microcirculatory changes that the oximeter measures concurrently. The circulatory system can be regarded as a pump system that pumps blood from the heart *via* the vasculature to each visceral organ. Any change in this system, including cardiac disease (pump dysfunction), vascular disease (circuit impairment), or renal obstruction(outflow stasis), may affect hemodynamic integrity and result in microcirculatory change [20].

Devarajan [21] suggested that serum creatinine should not be used as an indicator of rapid changes in kidney function because its concentration only accurately reflects kidney function in the steady state. Our comparison of patients with different grades of hydronephrosis indicated that only patients with grade I hydronephrosis had significant increases in eGFR at 14 days after URS. This indicates that the degree of hydronephrosis does not always correlate with renal function, and that eGFR may not be useful for evaluation of surgical success.

We reported the impact of changes in peripheral microcirculation induced by restoration of ureteral patency and urine flow as the percent change of PI (Table 3). The percent change of PI in the 3 hydronephrosis groups were not significantly different, suggesting no correlation between microcirculatory changes with hydronephrosis severity following stone disintegration. However, the percentage change of MAP negatively correlated with the percent change of PI (γ = −0.3, *p*<0.05). This may be because changes in microcirculation mainly affected the distal arterioles (which is innervated by the sympathetic system) and the smooth muscle tone in arterioles, and these preceded the observed change in MAP [22].

There can be great individual variations in PI, and several factors may interfere with measurement of PI [23], [24], so many previous studies used the pleth variabililty index (PVI) instead of PI to estimate volume status [25]–[27]. Nonetheless, our results suggest that PI can be used to assess changes in microcirculation in the perioperative period. The non-invasive nature of pulse oximetry makes it easy to use, and also makes it easy to gather information on changes in peripheral microcirculation [28], [29]. In addition, PI can indicate early changes in the sympathetic system and microperfusion in debilitated patients [30]. In this study, we successfully documented changes in microcirculation *via* PI following removal of ureteral obstructions. An animal study of the physiology of changes in hydronephrosis and relief of obstructive uropathy should be established to further examine the mechanisms of this effect.

In conclusion, our results showed that measurement of PI by a pulse oximeter allows monitoring of changes in peripheral microcirculation during endoscopic surgery for unilateral ureteral obstruction, but that eGFR did not change immediately after stone destruction. Use of a pulse oximeter to measure PI is simple and non-invasive, and provides important information regarding microperfusion in surgical patients. Thus, this method may improve patient safety and help clinicians to make important and prompt decisions while in the operating room.

## Supporting Information

S1 Txt **IRB protocol.**
(DOCX)

S2 Txt **IRB protocol in Chinese.**
(DOCX)

S3 Txt **CONSORT diagram.**
(DOC)

S4 Txt **STORBE checklist for cross-sectional study.**
(DOC)
