# Supplementary material for: Perfusion Index Derived from a Pulse Oximeter Can Detect Changes in Peripheral Microcirculation during Uretero-Renal-Scopy Stone Manipulation (URS-SM)
Source: PLoS One. 2014 Dec 26;9(12):e115743. doi: 10.1371/journal.pone.0115743 (PMC4277408; doi:10.1371/journal.pone.0115743)
Supplement: S1 Txt — IRB protocol. (DOCX) [file pone.0115743.s001.docx]

**Project title** (if applicable, use same title as externally-funded research):

Use perfusion index to detect microcirculation change in hydronephrosis patients during URS-SM

**Type of Project** :

Observational study

**Institute**

National Taiwan University Hospital Anesthesiology Department

National Taiwan University Hospital Urology Department

**Expected dates of study**

2012/9/1~2013/6/30

Basic Data

**Purpose of project**

Use non- invasive monitor-perfusion index to detect microcirculation change in hydronephrosis patients during URS-SM

**Abstract of project**

Urine patency after URS-SM in hydronephrosis patients will result in microcirculation change and could be detect by perfusion index derived from mashimo radical 7. We make correlation with eGFR with is as a predictor of renal function

Background

More emphasis was put in microcirculation in recent study. Microcirculation change during anesthesia can not be detected via blood pressure measurement or cardiac output monitor, but it may make impact in organ preservation and mortality.

The circulatory system can be regarded as a pump system that pumps from the heart via the vasculature to each visceral organ. Any change in this system, including cardiac disease (pump dysfunction), vascular disease (circuit impairment), or renal obstruction (outflow stasis) may affect hemodynamic integrity and result in microcirculatory change. Thus, we design this observational study.

Method

This is a prospective observational study. After approval of IRB, the study nurse will perform blood drawing and data collection

Inclusion criteria

18-80 y/o patient diagnosed as urolithiasis related hydronephrosis and need URS-SM for treatment during study period. ASA class was between I~III

Exclusion criteria

Patients with PAOD, poor cardiopulmonary function, morbid obesity, use vaso-active agents, and refused intravenous anesthesia

Protocol

1. We obtained written informed consent from patients to being included in this study at the pre-anesthesia visit on the day before surgery, and no pre-medications were given to the patient before entering the operating room. We also draw blood to estimate GFR. (sample 1)
2. After arrival at the OR, each patient was placed in a supine position and fitted with a non-invasive blood pressure cuff, 3-lead electrocardiogram, and pulse oximeter probe (Masimo Radical-7^®^; Masimo Corp., Irvine, CA, USA) was attached to the index finger on the ipsilateral side of intravenous catheter.
3. Perfusion index (PI) was continuously measured and recorded as an average of five consecutive data readings. Ambient temperature was set to 22 °C and intravenous fluid that had been warmed to 37 °C was infused at a rate of 500 ml/hr.
4. Anesthesia was administered as target-controlled infusion of propofol, and a blood concentration target was set at 5 μg/ml with fentanyl 1 μg/kg. Entropy values were maintained between 40 and 60 by increasing or decreasing the target concentration of 0.5 μg/ml to maintain a steady state of sedation.
5. We record the data till 15 mins after stone extraction
6. We draw blood as sample 2 next morning
7. We draw blood as sample 3 to estimate GFR at OPD 14 days after operation

Is this a multi-site study Single site

Is this applicable for fast –track review? No

Location of study? Operating room in 4^th^ floor in national Taiwan university hospital

IS there any ministry to review this study ? No

Responsibility of this study? The host of this project

Resource of funding ? No

Medication usage ? No

Item of this project? Monitors

Conlicts of interests ? No

Territory of this study ? microcirculation signals

Keywords Anesthesia, microcirculation, perfusion index

Study Category and Design

Mode of study?

Observational study

Purpose of study? Mode of observation?

Preventive Case study

Randomized? Data collection period?

Single group corss-section

Blindness? Specimen preserved ?

Opened Yes

Contrast ?

No

Mode of intervention ?

Single group

Risk of intervention ?

Safe

Is there interim analysis ?

No

Is there host guideline?

No

IS this study involved new drug, new devices, or new technique and the objects were fragile population>

No

Data of participants

Estimated participants

150

Age minimum 18 maximum 80

Inclusion criteria

18-80 y/o patient diagnosed as urolithiasis related hydronephrosis and need URS-SM for treatment during study period. ASA class was between I~III

Exclusion criteria

Patients with PAOD, poor cardiopulmonary function, morbid obesity, use vaso-active agents, and refused intravenous anesthesia

Period of following

14 days

Include patients by gender?

No

Perform pregnancy test ?

No

Include healthy patients?

No

Radiation exposure ?

No

Is this study involved gene reprogramming or bio-hazard?

No

Blood drawing ?

Yes

Amount of blood drawing 5 CC/ sample

Times of blood drawing 3 times

Total amount 15 cc
