# Supplementary material for: Perfusion Index Derived from a Pulse Oximeter Can Detect Changes in Peripheral Microcirculation during Uretero-Renal-Scopy Stone Manipulation (URS-SM)
Source: PLoS One. 2014 Dec 26;9(12):e115743. doi: 10.1371/journal.pone.0115743 (PMC4277408; doi:10.1371/journal.pone.0115743)
Supplement: S2 Txt — IRB protocol in Chinese. (DOCX) [file pone.0115743.s002.docx]

計畫中文名稱

以灌流指標(perfusion index)觀察腎水腫病人接受輸尿碎石術的微循環變化

計畫英文名稱

Use perfusion index to detect microcirculation change in hydronephrosis patients during URS-SM

計畫類別

觀察性研究

機構 部門

台大醫院 泌尿部

台大醫院 麻醉部

預計試驗開始日期 預計試驗結束日期

2012/9/1~ 2013/6/30

基本資料

研究計畫目的

本研究計畫是希望可以利用非侵入性的方式得到接受輸尿管碎石的病人產生的微循環變化, 並與eGFR做比較

研究計畫簡要說明

計畫摘要

利用mashimo radical 7所得到的perfusion index記錄hydronephrosis的病人在接受URS-SM手術前後因為urine patency所造成的microcirculation change，並與eGFR做為腎臟功能的指標,與其做correlation analysis

計畫背景

Microcirculation 在最近的研究中越來越被重視，因為在麻醉過程中一些些微的變化並沒有辦法在blood pressure或是cardiac output中被表現出來，然而這些微小變化，的確會對器官保存或是存活率造成影響。 Circulatory circuit是將循環視為一個整體,從心臟幫浦，到血管，到腎臟排出製造尿液, 在這過程中有任何的變化都可能導致微循環的改變。因此，我們設計了這個觀察性研究。

實施方法及進行步驟

1. 研究設計、資料收集

這是一個前瞻性的觀察性研究。在經過IRB approval後，抽血、檢體及資料收集由研究護理人員執行

1. 研究方法與原因

納入條件

研究對象以18-80 y/o 被診斷為urolithiasis related hydronephrosis需接受輸尿管碎石手術的病人, 且麻醉分級為ASAI~III

排除條件

有周邊血管病變，心肺功能不佳，重度肥胖，使用血管收縮劑，及無法接受靜脈麻醉的病人

方法

1. 在接受手術前一日解釋並填寫參與研究計畫受試者同意書，於病房抽血及收集sample以計算eGFR (smaple 1)
2. 於手術當日到達開刀房後，平躺5分鐘 記錄baseline BP, HR, SpO2, PI,及PVI
3. 持續每5分鐘量測vital sign，室溫設於22℃，輸液為500ml/hr
4. 使用proporfol指標控制靜脈麻醉，目標濃度為5 μg/ml，並根據entropy調整濃度。Entropy<40降低目標濃度0.5μg/ml， entropy >60增加目標濃度0.5μg/ml
5. 記錄數值到石頭打通後15 min
6. 於手術後隔天清晨進行抽血 (sample 2)
7. 於手術後14日約回門診, 並實行抽血 (sample 3)

1.多中心試驗類別 單一中心

是否符合簡易審查條件?* 是 ( 請 上傳 簡易審查檢核表 )

計畫執行地點* 台大醫院4樓開刀房及麻醉部

是否有需審查本計畫結果之主管機關* 否

研究計畫責任歸屬*

本試驗中心/計畫主持人

試驗經費贊助來源*

自籌(自行研究無經費補助)/

使用藥物或器材提供者

常規醫療處置

研究項目

其他, 生理訊號

計畫主持人如與本計畫試驗委託者有下列關係時，應揭露之，請勾選並說明(台大醫院請另檢附「顯著財務利益暨非財務關係申報表」

不適用

研究領域

麻醉生理訊號, 微循環

關鍵字

Anesthesia, microcirculation, perfusion index

計畫類別與設計

研究模式

觀察性研究

研究目的 觀察模式

?預防 病例研究

隨機分配 資料收集期

單一組別 橫斷性

盲性試驗 生物檢體保留

開放 無

對照組

無

介入模式

單組

研究評估結果

安全

研究計畫是否有期中分析(interim analysis)?

否

是否有主持人手冊?

否

研究計畫是否有資料安全性監測計畫/資料安全監測委員會(DSMP/DSMB)( 符合

醫療法第八條規範之「新藥、新醫療器材、新醫療技術」之人體試驗或研究對

象為易受傷害族群(未成年人、受刑人、原住民、孕婦、精神病人等)之介入性

試驗，或本院計畫主持人主導之多機構合作臨床試驗，或非屬前述但顯著超過

最小風險之臨床研究必須提出資料安全性監測計畫)*

否

受試者資料

受試者預定招募人數

本院共 150人

最小年齡 18 最大年齡 80

受試者納入條件

18~80 y/o 接受常規輸尿管碎石並使用靜脈麻醉的病人

受試者排除條件

血行動力學不穩定, 過度肥胖, 使用血管收縮劑, 動脈疾病

每位受試者預期之試驗期限或追蹤期間

14天

是否有依性別選擇受試者?

否

如試驗有影響胎兒之可能，此研究是否須執行懷孕檢測及避孕措施

否

本試驗是否有納入健康受試者

否

受試者是否主要包含下列易受傷害族群?

否

本試驗過程中受試者將接受輻射暴露

否

計畫是否涉及基因重組或操作具有生物危險性之微生物(第1-4級危險群)

否

取樣是否抽血* 是

每次抽血量 5 CC/次

抽血總次數 3 次

總共抽血量 15 CC
