# Supplementary material for: Perfusion Index Derived from a Pulse Oximeter Can Detect Changes in Peripheral Microcirculation during Uretero-Renal-Scopy Stone Manipulation (URS-SM)
Source: PLoS One. 2014 Dec 26;9(12):e115743. doi: 10.1371/journal.pone.0115743 (PMC4277408; doi:10.1371/journal.pone.0115743)
Supplement: S3 Txt — CONSORT diagram. (DOC) [file pone.0115743.s003.doc]

**CONSORT 2010 Flow Diagram**

**Allocation**

**Analysis**

**Follow-Up**

**Enrollment**

Assessed for eligibility (n=126)

Excluded (n=13 )

♦  Not meeting inclusion criteria (n=12)

♦  Declined to participate (n=1 )

♦  Other reasons (n= 0)

Analysed (n= )
♦ Excluded from analysis (give reasons) (n= )

Lost to follow-up (give reasons) (n= )

Discontinued intervention (give reasons) (n= )

Allocated to intervention (n= )

♦ Received allocated intervention (n= )

♦ Did not receive allocated intervention (give reasons) (n= )

Lost to follow-up (give reasons) (n= 0 )

Discontinued intervention (give reasons) (n= 0)

Allocated to intervention (n= 113 )

♦ Received allocated intervention (n= )

♦ Did not receive allocated intervention (give reasons) (n= )

Analysed (n=113 )
♦ Excluded from analysis (give reasons) (n=0)

Randomized (n= )
